# Osteopathy in Germany: attitudes, beliefs and handling among general practitioners – results of a nationwide cross-sectional questionnaire survey

**DOI:** 10.1186/s12875-021-01545-2

**Published:** 2021-10-07

**Authors:** Gordian L. Schmid, Jeremias Kluge, Tobias Deutsch, Anne-Kathrin Geier, Markus Bleckwenn, Susanne Unverzagt, Thomas Frese

**Affiliations:** 1 Department of General Practice, Medical Faculty, University of Leipzig, Philipp-Rosenthal-Str. 55, 04103 Leipzig, Germany; 2 Institute of General Practice and Family Medicine, Martin-Luther-University Halle-Wittenberg, Magdeburger Str. 8, 06112 Halle (Saale), Germany

**Keywords:** Osteopathic medicine, General practice, Osteopathic manipulative treatment, Complementary alternative medicine

## Abstract

**Background:**

Osteopathy is a type of complementary medicine based on specific manual techniques. In many countries, including Germany, the profession is not officially regulated, and evidence for the effectiveness of osteopathy is insufficient for most diseases. Nevertheless, many health insurances in Germany offer reimbursement for therapy costs, if osteopathy is recommended by a physician.

This cross-sectional survey of German general practitioners (GPs) explored beliefs and attitudes towards osteopathic medicine and described their daily interactions with it.

**Methods:**

A random sample of 1000 GPs from all federal states was surveyed by mail using a self-designed questionnaire. We collected data on sociodemographics, personal experiences with osteopathy, and attitudes and expectations towards osteopathy. In particular, participants were asked about indications for osteopathic treatment and their beliefs about its effectiveness for different patient groups and diagnoses. A self-designed score was used to estimate general attitudes towards osteopathy and identify factors correlated with greater openness. Additionally, we performed logistic regression to reveal factors associated with the frequency of recommending osteopathy to patients.

**Results:**

Response rate was 34.4%. 46.5% of participants were women, and the median age was 56.0 years. 91.3% of GPs had referred patients to an osteopath, and 88.0% had recommended osteopathy to their patients. However, 57.5% acknowledged having little or no knowledge about osteopathy. Most frequent reasons for a recommendation were spinal column disorders (46.2%), other complaints of the musculoskeletal system (18.2%) and headaches (9.8%). GPs estimated the highest benefit for chronically ill and middle-aged adults. Female gender (OR 2.09; 95%CI 1.29–3.38) and personal treatment experiences (OR 5.14; 95%CI 2.72–9.72) were independently positively associated with more frequent treatment recommendation.

**Conclusion:**

GPs in Germany have frequent contact with osteopathy, and the vast majority have recommended osteopathic treatment to some extent in their practice, with foci and opinions comparable to other Western countries. The discrepancy between GPs making frequent referrals for osteopathic treatment while self-assessing to have little knowledge about it demonstrates need for intensified research on the collaboration with osteopaths and how to best integrate osteopathic treatments. Our results may help to focus future effectiveness studies on most relevant clinical conditions in general practice.

**Supplementary Information:**

The online version contains supplementary material available at 10.1186/s12875-021-01545-2.

## Background

Osteopathy, also referred to as osteopathic medicine or osteopathic manipulative treatment (OMT), is a complementary medicine with its own philosophy, methods of diagnosis and manual therapy [1]. Focus is placed on the human body unity, determined by autoregulation and the interactions of anatomy and physiological function. The treatment method was founded by US-American physician and preacher Andrew Taylor Still in the late nineteenth century and spread from there to Europe and many other parts of the world [2].

The regulation of osteopaths is diverse among European countries. While in Denmark, Finland, France, Iceland, Lichtenstein, Malta, Portugal, Switzerland and the UK osteopathy is approved and regulated by the state [3–5], in Germany there is no legally protected professional title and no uniform training or curriculum exists. Nevertheless, with around 10,000 osteopaths and more than 10 million patient contacts per year, osteopathy is an recognizable economic and financial part of the health sector in Germany [6]. Osteopathy in Germany legally has been defined as a medicine system that can only be applied legally by physicians or state-approved alternative practitioners (“Heilpraktiker”). However, osteopathy is not accredited as official additional training for physicians in Germany. Physiotherapists may only treat patients with osteopathic techniques when prescribed by a physician, although they cover a large part of the osteopathic treatment [7]. Osteopathic training in Germany mostly takes place at private schools or universities that are partially and voluntarily supervised through associations of osteopaths or alternative practitioners. The curricula and the extent of training hours show a high variation among schools [1].

Evidence for the effectiveness of osteopathic treatment is scarce for most indications not directly related to musculoskeletal problems of the spine (e.g., low backpain) and often has methodological problems [1, 8].

Many German health insurance organisations reimburse the costs for osteopathic treatment partially, if a physician recommends this kind of treatment [9]. General practitioners (GPs) are often the first professional contact for patients seeking osteopathic treatments. That’s why patients enquire them with questions towards the indications, effectiveness and safety. However, little data and knowledge of the relationship and views of German GPs on osteopathy is available.

The present study explored the knowledge, beliefs and attitudes among German GPs towards osteopathy, as well as their experience with osteopathy in practice. The study also aimed to identify associations between sociodemographic, job-related and experience-based variables which could influence the treatment recommendation for osteopathy. In addition, reasons for referral and expectations of benefit for selected patient groups and treatment occasions were investigated.

## Methods

### Sampling and design

A cross-sectional survey of a random sample of 1000 GPs across Germany was performed. The sample size was calculated based on a number of 45,467 GPs working in Germany in 2019 [10], aiming for a level of confidence of 95% and a precision of 5%. Due to the controversial topic, we assumed a response rate lower than average of 30 to 40% [11]. So, we decided to contact 1000 GPs by mail. Numbers of selected GPs for each federal state were balanced to the number of respective practicing GPs based on 2018 figures of the Associations of Statutory Health Insurance Physicians (Kassenärztliche Vereinigungen) [12, 13]. From 200 randomly chosen postal codes, five GPs having their practice in the respective area were selected by random draw from publicly available registers. If there were fewer than five GPs registered, the missing addresses were taken from another random postal code of the same state.

**Fig. 1:**
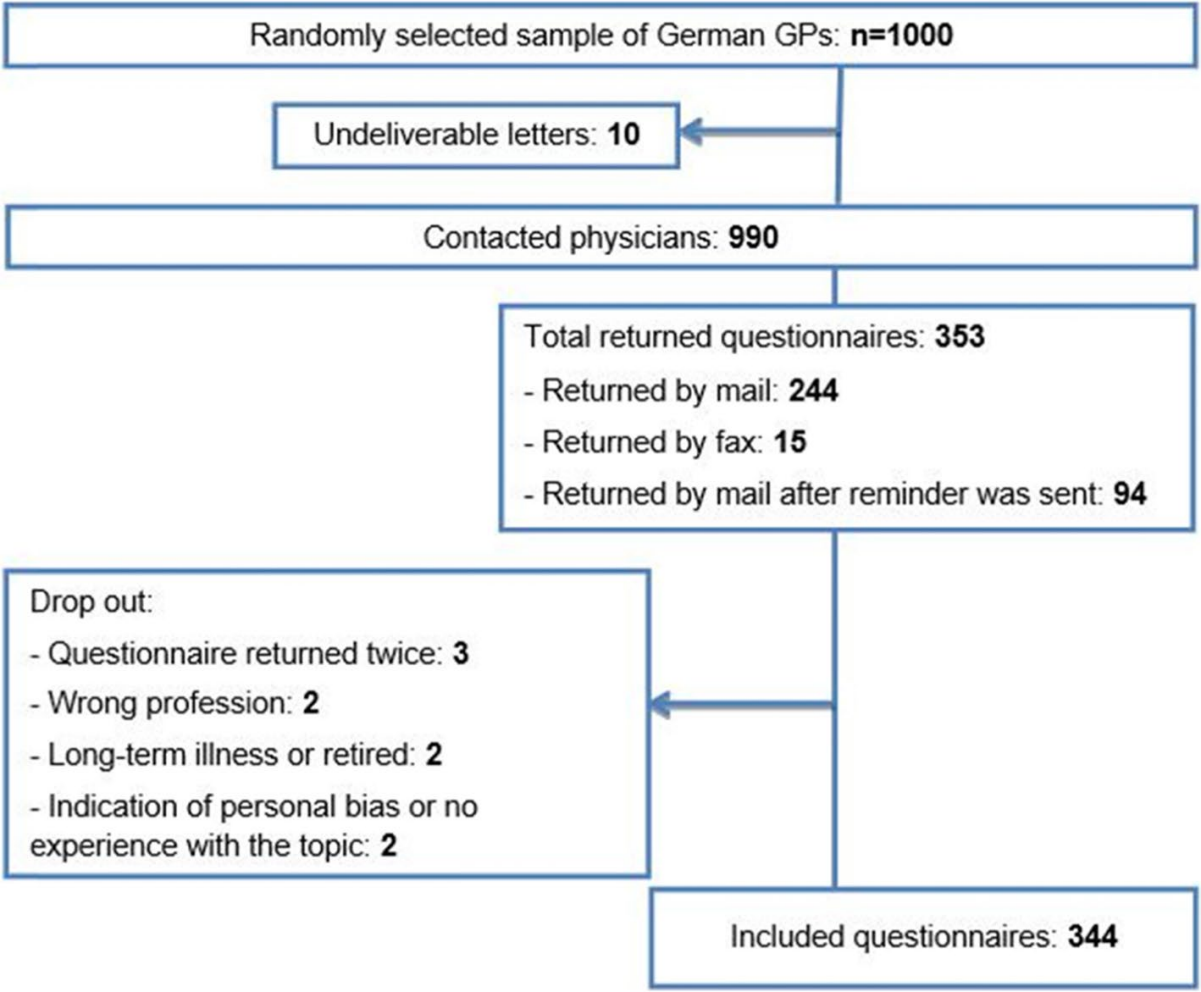
Sampling flow chart

In February 2019, selected GPs were mailed the questionnaire, as well as a formal cover letter containing information about the study and a privacy statement. After 2 months, a reminder was sent. No incentives were offered. The survey was closed for evaluation in July 2019. Participation in the study was voluntary, and completed questionnaires were returned by fax or mail. The detailed sampling process is shown in Fig. 1.

### Questionnaire

The questionnaire was self-designed with the input and expertise of an interdisciplinary research team consisting of experienced GPs and physicians (one with additional expertise in osteopathy), a social scientist and a medical student. An unsystematic literature search using the keyword “osteopathic treatment” was performed in Medline/Pubmed and the Cochrane Library considering the number of publications found for the most common presenting complaints. Positive or negative effect ratings were not considered. This search identified 15 common conditions, that were most frequently addressed in the identified studies. Those conditions should be rated by the participants regarding expected benefit of osteopathic treatment. To ensure comprehensibility and face validity, the questionnaire was pre-tested by 8 GPs, including subsequent feedback discussions. After minor modifications, the final version contained 55 items and could be completed in 5 to 10 min. An English translation of the questionnaire is provided in Additional file 1.

### Data collection and statistical analyses

Returned questionnaires were scanned and read out using the software Form Pro 3.0 (OCR Systems, Germany). Collected data was cleaned, and the free text answers were added manually. Statistical analyses were performed using IBM SPSS Statistics 24 for Windows. Frequencies were presented as %_valid_ (n_absolute_/n_valid_), continuous variables as mean ± standard deviation (SD). We categorized the open text diagnoses into subcategories with different levels of specification, depending on related clinical pictures. The outcome variable and key question “Have you ever recommended treatment by an osteopath to a patient?” was coded binary (0 = in a few cases/no, never; 1 = yes, regularly/occasionally). The response options were subjective without numerical specifications for the frequency of recommendation. Univariable and multivariable binary logistic regression was used as the primary analysis to estimate predictive factors of recommendation behavior (dependent variable) including 95% confidence intervals (95% CI). After selecting variables based on content to include in the analysis, individual tests of the items to exclude multicollinearity were undertaken. Reasons for exclusion were correlation of two variables r > 0.6, variable categories with a total of *n* < 10 respondents, generally high rate of missing answers for single questions (*n* < 300) and *p* > 0.5 in the following Chi-square tests. Covariates gender (men vs. women), age (< 40 vs. ≥ 40), personal treatment experience (yes vs. no) and knowledge (very good/good vs. little/no) were investigated as independent predictor variables. In an exploratory approach, backward LR/stepwise and forward LR/stepwise sensitivity analyses were performed.

To estimate the general attitude and the subjective appraisal towards osteopathy, we used five questions of the questionnaire and created a score to summarize this general view of GPs. For each question, a value between 0 (rather skeptical) and 3 (rather open-minded) was assigned to generate a total score. A higher score represents a more positive attitude towards osteopathy. Linear regression (stepwise) explored the relationship between this score and selected predictor variables as above.

## Results

### Descriptive analysis

#### Response

The response rate was 34.4%, with 344 (of 1000) analyzable data sets. Figure 1 shows a flow chart with details about returned questionnaires and reasons for exclusion. Response rate was heterogeneous, with the highest rates in the federal states Saxony, Schleswig-Holstein and Thuringia (44.6 to 50.0%) and lowest rates in Hessen (24.6%) and North Rhine Westphalia (26.2%).

#### Sociodemographics and job-related characteristics

Of the respondents, 46.5% were women. The mean age was 54 ± 9.5 years (Median 56.0, range 30–80). 64.5% of the GPs had at least one additional qualification, while only 4 out of 344 (1.2%) had extensive training in osteopathy. Table 1 shows a summary of sociodemographics and comparable data of all German GPs, available from publicly accessible registers [14].

**Table 1:** Sociodemographic and job-related factors - total sample compared to all German GPs in %

**Variable**	**All participating GPs in % (n/nvalid)**	**All German GPs in %**
Female	46.5 (160/344)	45.9
Age in years (mean ± SD)	54.8 ± 9.5, Median: 56.0	55.5
≤ 39 years old	6.8 (23/340)	6.3
40-49y	20.6 (70/340)	20.4
50-59y	39.7 (135/340)	37.2
60-65y	19.4 (66/340)	20.1
> 65y	13.5 (46/340)	15.9
Has a doctor’s degree or habilitation	64.3 (205/319)	
Training in Osteopathy	1.2 (4/344)	
Completed at least one additional qualification	64.5 (222/344)	a
Manual medicine/Chiropractic/Physical medicine	19.5 (67/344)	8.4
Emergency medicine	13.7 (47/344)	8.1
Acupuncture	12.8 (44/344)	7.3
Psychotherapy/Psychosomatics	9.0 (31/344)	7.4
Homeopathy	8.7 (30/344)	3.0
Sports medicine	8,4 (29/344)	6.1
Working in own practice (versus employed)	85.7 (288/336)	79.7
Years having own practice (mean ± SD, nvalid = 286)	20 ± 10.6	
Legal structure of the practice		
Single practice	58.5 (158/270)	55.7
Joint practice	37.8 (102/270)	39.0
Medical care center (“MVZ”)	3.7 (10/270)	5.3
Practice environment (self-assessment)		
Big city	22.1 (73/330)	
Small city	37.6 (124/330)	
Countryside	40.3 (133/330)	
State where doctor is working		
States of former East Germany + Berlin	24.5 (68 + 16/343)	20.8
States of former West Germany	75.5 (259/343)	79.2

^a^Percentage refers to all physicians in outpatient settings, not only GPs (data not separately available)

#### Handling of osteopathy in daily practice

Of all respondent GPs, 33.7% reported recommending osteopathy frequently, 35.8% occasionally, 18.5% in a few cases and 12.0% never. Nevertheless, almost every GP (91.3%) had already given a written recommendation or referral for osteopathic treatment to patients. In most cases, this recommendation happened partly on their own suggestion and partly following the patient’s request (64.6%). In 29.9% of cases, the referral occurred on the patient’s request only, and in 5.4% of cases, on the physicians’ suggestion alone. If the physicians reported rejecting making a formal recommendation for patients (9.3%), the major reasons were lack of trust/evidence (40.0%) and that they considered other therapies to be better or more effective (24.0%).

The majority of GPs surveyed (77.6%) personally knew an osteopath working in the catchment area of their practice, and of these GPs, 57.1% had collaborated with them. The questions addressing cooperation quality revealed that 42.9% of GPs exchanged medical findings with their local osteopath. The majority of GPs considered the information exchanged comprehensible (74.2%) and useful for treatment (64.9%). Willingness to cooperate with osteopaths was present in two-thirds (67.8%) of the doctors. More than one-third of GPs (39.4%) received patients sent by an osteopath. Furthermore, 33.7% of all GPs had been treated by an osteopath themselves. Almost two-thirds (66.1%) knew a qualified osteopath whom they would recommend to patients.

When asked about the general feedback given by patients after osteopathic treatment, 69.0% of doctors reported receiving overall positive feedback. 22.7% of respondents indicated they received heterogeneous feedback, and only 2.7% emphasized that they received negative feedback. 5.7% of GPs indicated they did not receive patient feedback after osteopathic treatment.

#### Knowledge

Reported subjective responses on knowledge were widely divergent, from self-perceived very well-informed (7.6%) and good knowledge (34.6%) to little (52.6%) and almost no expertise (4.4%) in osteopathy. From *n* = 628 submitted replies (multiple answers were possible), personal narratives (38.1%) and medical journals (28.2%) were the highest rated sources of information. Internet (7.8%), non-medical journals (5.1%), training/further education or congresses (4.1%) and exchange with colleagues or patients (2.9%) followed. Additionally, 17.7% of GPs stated that they have little or no information on the subject of osteopathy at all.

Nearly three-quarters (72.2%) of GPs were aware of which medical professions are allowed to practice osteopathy in Germany.

#### Treatment occasions and patient spectrum

**Fig. 2:**
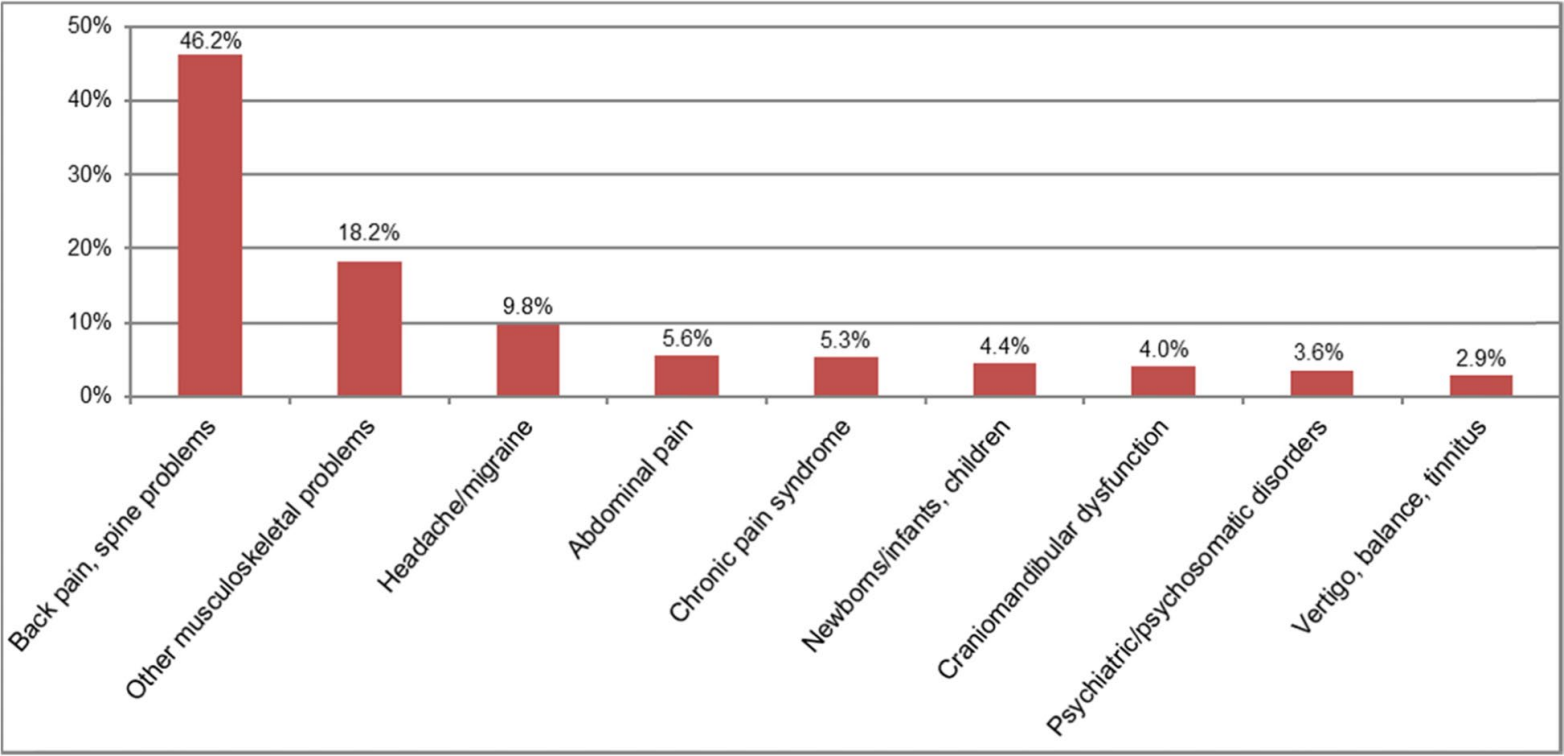
Open text reasons for encounter of most likely recommended clinical pictures for osteopathic treatment in % (*n* = 450)

When asked for the most frequent reason for recommendation of osteopathy, in total 629 reasons for encounter were mentioned and clustered into nine categories. The most frequent reasons listed were back pain and complaints concerning the spinal column (cervical/thoracic/lumbar spine, sacroiliac joint, discus prolapse). Other treatment occasions that could not be assigned to any of the selected categories (*n* = 20; e.g., dysmenorrhea, scar pain, post-traumatic or postoperative treatment, insomnia, heart complaints) are not mentioned in the detailed percentages (Fig. 2).

**Fig. 3:**
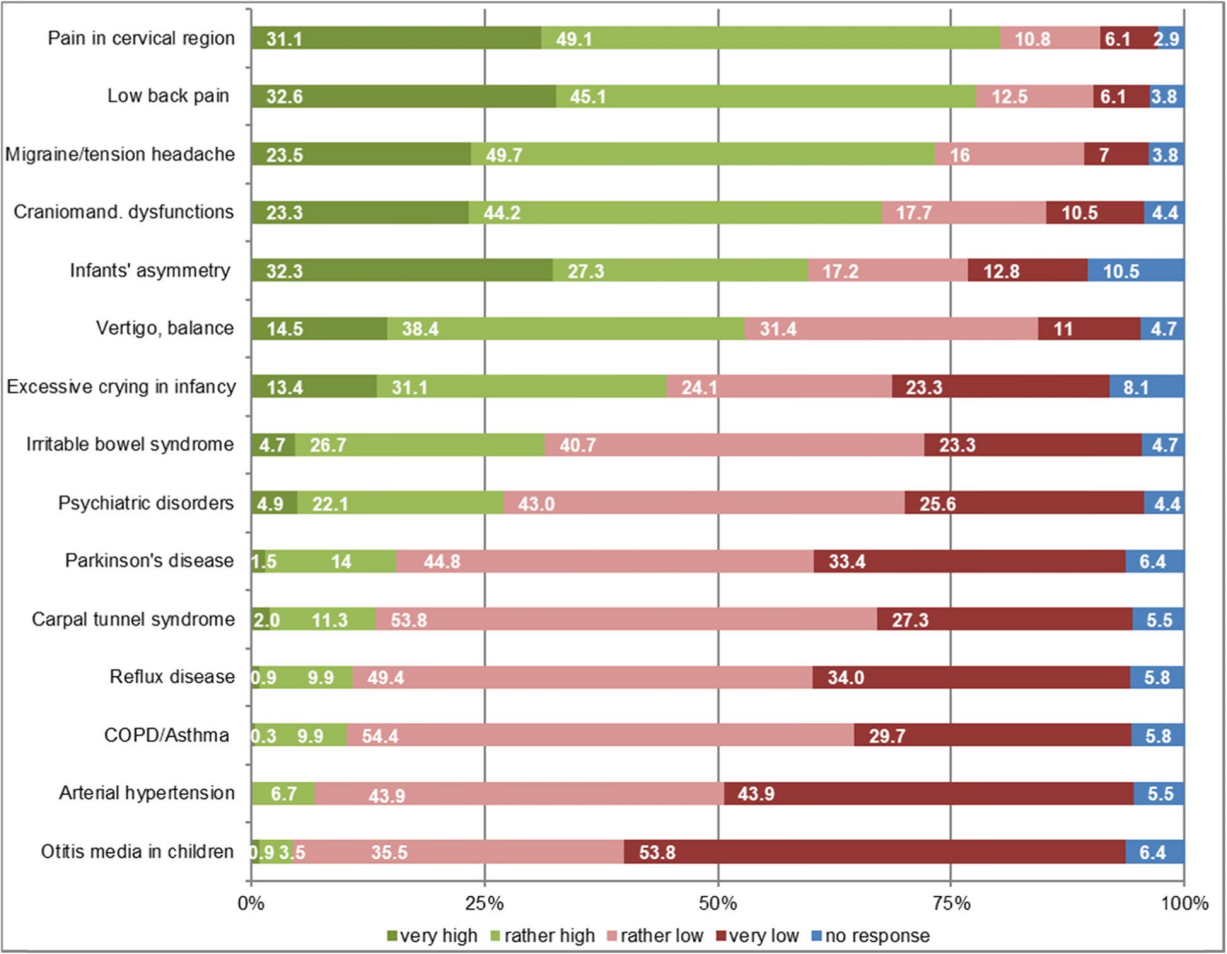
Expectations of benefit from osteopathic treatment for different pre-selected reasons for encounter via GPs self-assessment in % (*n* = 344)

**Fig. 4:**
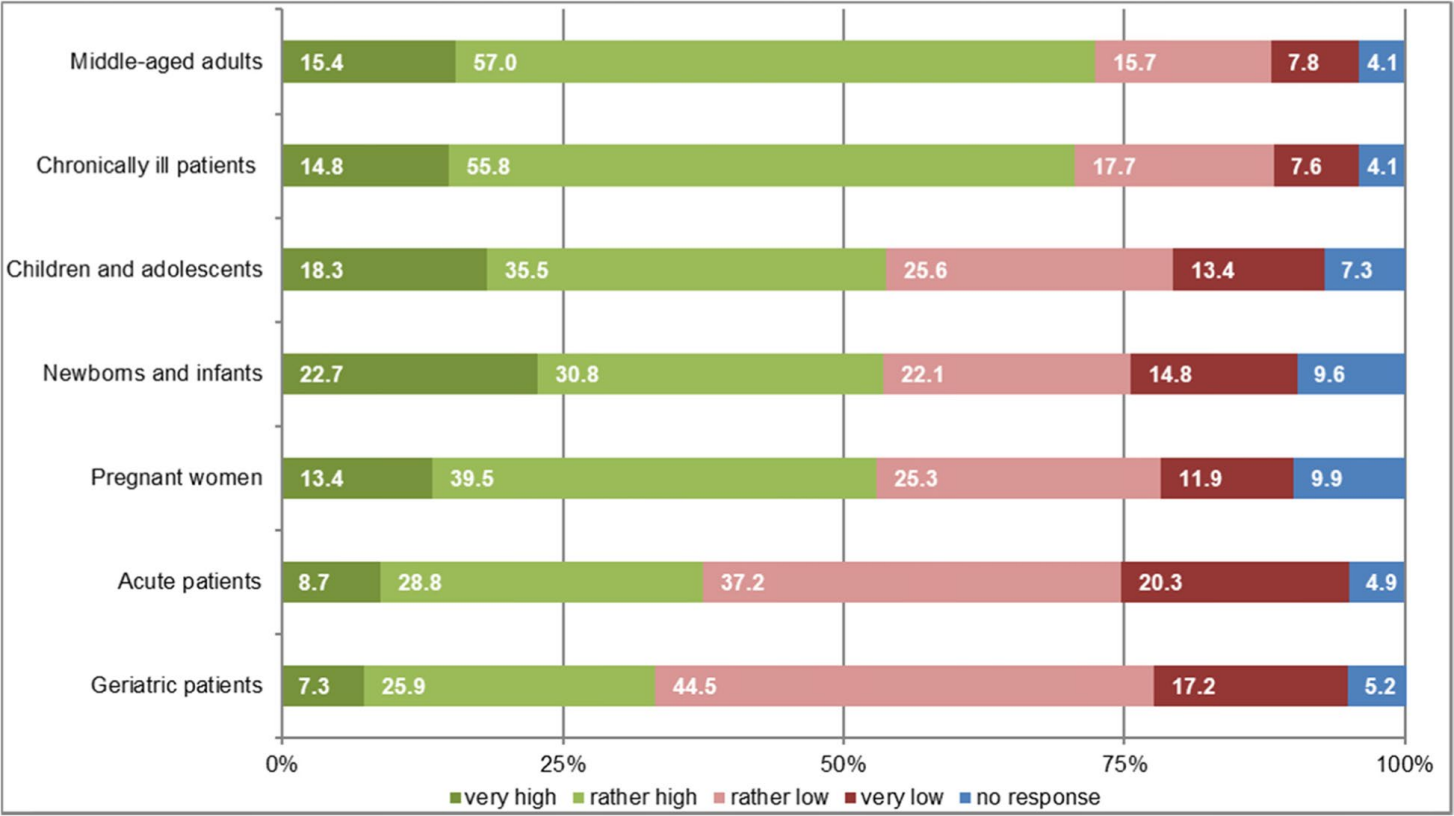
Expectations of benefit from osteopathic treatment for different patient groups via GPs self-assessment in % (*n* = 344)

Asked about the treatment occasions for which GPs prefer to refer patients to osteopaths, physicians rated musculoskeletal causes the highest, in line with the open-text answers. Complaints associated with the inner organs were faced with lower expectations (Fig. 3). Middle-aged and chronically ill patients were attributed the biggest benefit for OMT (Fig. 4). Female GPs had a significantly higher expectation of benefit assessment than male GPs in 9 of 15 clinical pictures and 4 of 7 patient groups.

#### Opinions on scientific and political issues of osteopathy

**Fig. 5:**
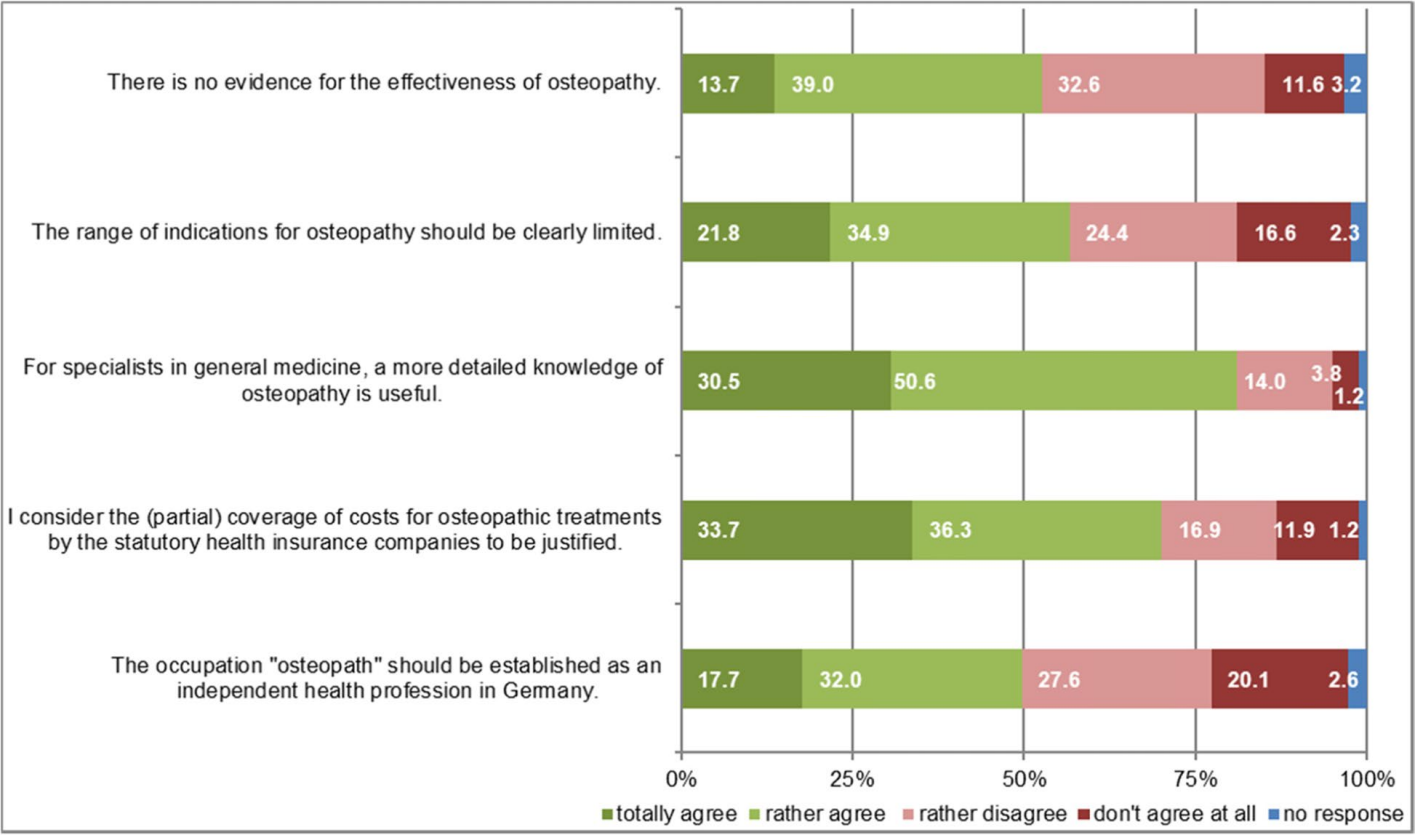
“How do you agree with the following statements?” answers in % from all GPs (*n* = 344)

GPs were asked about five current issues of debate concerning osteopathy in respect to the evidence base, need for information, reimbursement by health insurance companies, limitation of indications and establishment of a distinct health profession (Fig. 5).

### Opinion score

With *n* = 321 GP included, we have scored the “opinion score” by five questionnaire items with a standard distributed outcome (mean value 8.3 points ± 3.5, range 0–15). This serves as an attempt to represent approval and rejection of the treatment method osteopathy. No multicollinearity (> 0.7) could be detected between the five questions.

When correlating the items already examined in the cross table with the opinion score, a clear association with gender (*p* = 0.004), knowledge, patient feedback, self-treatment, and knowing a locally based and qualified osteopath (all *p* < 0.001) for a higher opinion score was shown. Correlation to age, additional qualification and the federal state could not be found.

Notably, multivariate regression showed higher attitude score in physicians with former self-treatment (*p* < 0.001; regression coefficient 2.78, 95%CI 1.97–3.57). GPs, who have already been treated themselves, are more open-minded towards osteopathy. State and gender also remained as influencing variables.

### Associations with treatment recommendations

Results of bivariate associations are presented as absolute and relative numbers from the selected items in Additional file 2. Female practitioners (OR 2.09; 95%CI 1.29–3.38) more frequently recommended osteopathic treatment. GPs who know a local (OR 4.11; 95%CI 2.43–6.96) and qualified (OR 6.94; 95%CI 4.14–11.64) osteopath recommended OMT more often, as well as GPs who have been treated themselves (OR 5.14; 95%CI 2.72–9.72). Furthermore, more frequent treatment recommendation was positively associated with good feedback from patients (OR 7.26; 95%CI 4.31–12.22) and knowledge of the GP about osteopathy (OR 1.91; 95%CI 1.17–3.10). No significant association could be found with age, additional qualification, or catchment area and structure of the practice.

Four covariates were preselected for multivariate analyses and analyzed on the basis of 302 questionnaires (87.8% of all GPs) with complete answers. Multivariable analysis predicting self-assessed occasionally or regularly (vs. few cases or no, never) recommendation of osteopathy revealed positive associations for physicians with own treatment experience (88.3% vs. 59.5%; OR 5.44; 95% CI 2.77–10.70). Furthermore, female physicians seem to prefer osteopathic treatment (77.7% vs. 62.5%; OR 1.62; 95% CI 0.94–2.82). These results were confirmed in the sensitivity analysis.

### Responder and non-responder analyses

No difference was stated between response and gender (*p* = 0.229), GPs practicing in former Eastern or Western Germany (*p* = 0.094) and academic degree of GPs (see Table 1).

Non-response rate among female and male GPs was 63.8 and 67.0%, respectively. Considering GPs in former Eastern and Western Germany, this rate was 56.9 and 67.8%. There was no difference in the frequency of academic degree for responders (64.3%) and non-responders (64.0%).

## Discussion

Osteopathy seems to be a well-known topic for German GPs, as nearly all respondents had already given written recommendation for an osteopathic treatment to their patients. More than three-quarters of all respondents knew an osteopath near their practice, two-thirds could imagine cooperation with osteopaths, almost two-thirds could name an osteopath they would send patients to and more than half of the GPs already exchanged information with an osteopath about patients treated together. Similar rates of treatment recommendations were also given in other publications [15–21]. A study among GPs in London found that osteopathy was the most common referral among treatments categorized as Complementary Alternative Medicine (CAM). 84% of these physicians had received requests from patients for a referral, and 78% had already suggested a referral for osteopathy [18]. Among British GPs, 9.1% referred and 31% endorsed OMT within the last week [15]. In an Australian survey, 63% of GPs referred patients to osteopaths and chiropractors at least a few times per year [21]. A further Australian study from 2005 showed a lower recommendation rate for osteopathy of 23% within the last 12 months [20]. Active physicians’ recommendation for CAM plays a significant role in the patient’s appropriate use, perception and evaluation of the therapy [22, 23]; therefore, the GP has a significant influence on the patient’s decision making. When comparing our data with studies from other western countries, it is important to note that the historical development of osteopathy has taken place at different speeds and depths. Additionally, the allocation of osteopathy to the respective national health system is multifaceted.

Despite the regular and diverse points of contact, more than half of the physicians surveyed have little or no knowledge about osteopathy itself. Personal narratives were cited by 69.5% as one source of information, and more than one-quarter did not know which persons are legally justified to apply osteopathic treatments. Those facts reveal a remarkable lack of information among German GPs. Comparable percentages were given in the UK, with 60% of GPs defining themselves as having little confidence in their osteopathic knowledge [15]. Other studies from the Australia and Canada showed similar numbers [19, 24]. However, it seems that British GPs were significantly better informed about official qualifications of osteopathic practitioners than hospital doctors, and 84% of these GPs at least knew the main principles of osteopathy [18].

Diagnoses concerning the spine and other musculoskeletal locations made up more than 60% of the free text diagnoses. In the expectations of benefit given by GPs, low back pain, cervical/neck complaints and headache/migraine were named most frequently. This is in line with the comparatively high amount of external evidence on osteopathic manipulative treatment for (lower) back pain [25–28]. In addition, our findings are also in line with the most common specific complaints evaluated in Benelux Osteosurvey 2013 with osteopathically self-evaluated treatment occasions [4]. They were mostly focused on musculoskeletal and spinal column-related conditions. For 81% of patients in the Swiss Osteosurvey 2018, musculoskeletal pain mainly located in the cervical, lumbar and fascial area played a role in the decision towards OMT [29]. Meta-analyses of a Spanish osteopathic patient profile showed lumbar and cervical diagnoses as well as headaches as the main reasons for a consultation [30]. Infants’ asymmetry played a minor role. Some special groups of patients (e.g., children, pregnant women), which are often described in effectiveness studies of OMT [31, 32] as well as clinical conditions (e.g., infant asymmetry or excessive crying) are rather seldom present in German GP practices and mostly treated by specialists. This might be a reason for the low numbers of recommendations for those patients in this study.

Women in the current study are more likely to be convinced about osteopathy across almost all items of the questionnaire and give a better benefit rating. This is in line with the German government’s health report on CAM from 2002 which noted that women are more open to the use of unconventional treatment methods in general [33]. Women use alternative treatment methods significantly more often than men [34, 35]. This finding is therefore not specific for our study and osteopathy but rather generally valid for women and CAM.

If the physician knows an osteopath who is qualified and, at best, lives nearby, if the patients’ feedback is positive and if the GP has even been treated himself/herself, the recommendation rate of therapy to patients is significantly higher. This association was also found in other studies [36–38] and is in line with results from a study performed among Estonian GPs in 2007. GPs who have been treated themselves and who have greater belief in the effectiveness and evidence base for osteopathy more often refer to osteopaths [39]. In the UK study, the lowest 10% scores for estimated effectiveness of osteopathy were given by GPs who were male, over 50 years old, and/or working in a single practice [15]. In contrast, an Australian survey from 2013 showed significant associations for osteopathic referral with knowledge about osteopathy, patient load per week, own experience with CAM, patients asking and request for referral, positive feedback from patients and belief in the efficacy of osteopathy. Demographic factors like age, gender, level of rurality and location of medical school were not predictive for referral to osteopaths [24]. Contrary to our findings, there was no significant difference found between female and male GPs referring chiropractic/osteopathic treatment in an US-American study [16].

Our questionnaire contained five items on GPs’ opinions and views towards osteopathy. Approximately a balanced number of positive and negative opinions among GPs was observed regarding establishing an independent osteopathic profession, an overall lack of sufficient evidence for osteopathy and a demand to limit osteopathic treatment indications. Three-quarters favored having costs covered by health insurances and supported more knowledge about osteopathy for GPs. Thus, the topic seems to polarize. Critical opinions are partly in contrast to the widespread practical use of osteopathy. The opinions in other western countries diverge widely. OMT was rated in comparable international surveys as useful and effective among 34 to 50% of GPs [15, 21, 24]. In contrary, 50% of Australian GPs assessed osteopathic education as not primarily evidence based [19]. Osteopathy as part of the health care system would be accepted by 45.2% of GPs in Estonia [39]. In the UK, 52% of GPs indicated that the NHS should pay for this therapy [15]. Moreover, 91% of GPs agreed all osteopathic practitioners should be formally qualified and licensed by law [18]. The questions of evidence, political recognition and regulation of indications for OMT remain to be clarified in Germany.

### Strengths and limitations

Regarding the link between GPs and osteopaths, our cross-sectional study is to our knowledge the first of its kind in Germany. The sample is balanced throughout the whole country with a reasonable sample size, and the substantial response rate supports the explanatory power of our findings.

We cannot exclude or definitively determine the size of a selection bias due to interest in the topic and a positive view towards CAM and osteopathy. Social desirability might also have influenced response rates.

In the questionnaire, free text diagnoses and unspecific answers complicated a precise categorization of some diagnoses.

To gauge general attitudes towards osteopathy, we used a set of five items rather than a single direct question. We asked indirectly and included several dimensions of opinion. The proposed score is based on self-designed items and has not been validated. A direct question like “How much do you sympathize with osteopathy in general?” may have been helpful. Furthermore, we figured out the problem of causal relationship and the complete exclusion of multicollinearity as difficult to solve. We could not distinguish whether a GP was first self-treated and then recommended OMT to patients, or vice versa.

## Conclusion and implications for practice

Osteopathy is frequently recommended in general practices in Germany, more often among female GPs and physicians having their own previous experience with osteopathy. Most frequent reasons for a recommendation are disorders of the spinal column followed by other complaints of the musculoskeletal system and headaches. This study can provide the basis and orientation for future research on patients’ needs and efficacy of osteopathic treatment. Nevertheless, there is a lack of information among German GPs. Targeted, concise information material or guidelines [2] about the philosophy, treatment methods, risks, scientifically evidence base and the legal situation could support GPs in their function as health-care adviser dealing with osteopathy and may lead to the safe and well-informed use of this treatment for patients.

## Supplementary Information

**Additional file 1.** English translation of the questionnaire (original language: German).

**Additional file 2.** Cross table with univariate predictors of recommendations (all items matched, ntotal = 341).

## Data Availability

The datasets used and analyzed during the current study are available from the corresponding author on reasonable request. Open Access funding enabled and organized by Projekt DEAL.

## Abbreviations

CAM: Complementary Alternative Medicine
CMD: Craniomandibular Dysfunction
COPD: Chronic obstructive pulmonary disease
GP: General practitioner
OMT: Osteopathic manipulative treatment
