# Supplementary material for: Osteopathy in Germany: attitudes, beliefs and handling among general practitioners – results of a nationwide cross-sectional questionnaire survey
Source: BMC Fam Pract. 2021 Oct 7;22:197. doi: 10.1186/s12875-021-01545-2 (PMC8499418; doi:10.1186/s12875-021-01545-2)
Supplement: Supplementary file 1 — Additional file 1. English translation of the questionnaire (original language: German). [file 12875_2021_1545_MOESM1_ESM.pdf]

(1) English translation of the questionnaire (original language: German)

UNIVERSITÄT LEIPZIG

Selbstständige Abteilung für Allgemeinmedizin

Fax + 49 341 97-28309

Osteopathy in Germany: attitudes, beliefs and handling among General Practitioners

IMPORTANT: In order for us to include your questionnaire in our study, it is important that all questions are answered completely.
Thank you!

Personal details and medical practice

Age: _____ Gender: ○ female ○ male

At which university (/-ies) did you study human medicine? ______________________________________

Highest academic qualification: ○ State examination ○ Diploma ○ Promotion ○ Habilitation

Acquired specialist titles (please name all of them and indicate the year of graduation)

Specialist for ____, since: _______ (year)

Specialist for ____, since: _______ (year)

Acquired additional qualifications (please name all)

____

____

Type of current work: ○ employed, since: _______ (year) ○ self-employed/own practice, since: _______ (year)

Legal structure of the practice: ○ Single practice ○ Joint practice ○ Medical care center (MVZ)

How would you most likely describe the catchment area of the practice? ○ big city ○ small city ○ countryside

Experience and handling of osteopathy

Have you ever recommended patients to be treated by an osteopath?
○ yes, regularly ○ occasionally ○ in a few cases ○ no, never

Have you ever given patients a private prescription/recommendation for osteopathic treatment? ○ yes ○ no

If yes: ○ at my own suggestion ○ at patient's request ○ both

If yes: For which diagnoses do you prefer to refer to an osteopath for treatment? __________________________

__________________________ __________________________ __________________________

If no, why not? ____

Do you know osteopaths who work in the catchment area of your practice? ○ yes ○ no -> continue to **B**

If yes: has there been any cooperation with them? ○ yes -> continue to **A** ○ no -> continue to **B**

A Assessment on the common treatment of patients:

There was an exchange of findings.	○ yes	○ rather yes	○ rather not	○ no
The information from the osteopath was understandable.	○ yes	○ rather yes	○ rather not	○ no
The communication with the osteopath was useful for the treatment.	○ yes	○ rather yes	○ rather not	○ no

B Would you like to work /cooperate with osteopaths? ○ yes ○ rather yes ○ rather not ○ no

Have patients ever been sent to your practice by an osteopath? ○ yes ○ no

Do you know a qualified osteopath whom you would recommend to patients? ○ yes ○ no

Have you ever been treated by an osteopath yourself? ○ yes ○ no

What general feedback do patients give you on average after osteopathic treatment?
○ mainly positive ○ rather positive ○ heterogeneous ○ rather negative ○ mainly negative ○ no feedback

How do you estimate the benefit of osteopathic treatment for the following patient groups?

	Very high	Rather high	Rather low	Very low
Newborns and infants	○	○	○	○
Children and adolescents	○	○	○	○
Pregnant women	○	○	○	○
Middle-aged adults	○	○	○	○
Geriatric patients	○	○	○	○
Acute patients	○	○	○	○
Chronically ill patients	○	○	○	○

How do you estimate the benefit of osteopathic treatment for the following clinical pictures?

	Very high	Rather high	Rather low	Very low
Low back pain	○	○	○	○
Migraine/tension headache	○	○	○	○
Infants' asymmetry	○	○	○	○
Pain in the cervical region	○	○	○	○
Vertigo, balance problems	○	○	○	○
Obstructive lung diseases	○	○	○	○
Temporomandibular dysfunctions	○	○	○	○
Carpal tunnel syndrome	○	○	○	○
Irritable bowel syndrome	○	○	○	○
Gastroesophageal reflux disease	○	○	○	○
Arterial hypertension	○	○	○	○
Psychiatric disorders	○	○	○	○
Parkinson's disease	○	○	○	○
Otitis media in children	○	○	○	○
Excessive crying in infancy	○	○	○	○

Where do you get information about osteopathy? (multiple answers possible)

○ Medical journals ○ Non-medical journals ○ personal stories
○ Television ○ Internet ○ Other:_______________ ○ have hardly/no information on the subject

How would you rate your knowledge of the concepts of osteopathy and its treatment techniques?
○ very good knowledge ○ good knowledge ○ little knowledge ○ no knowledge

Did you know that osteopathy in Germany may only be practiced by doctors and alternative practitioners? ○ yes ○ no

**How do you agree with the following statements?**	Totally agree	Rather agree	Rather disagree	Don't agree at all
There is no evidence for the effectiveness of osteopathy.	○	○	○	○
The range of indications for osteopathy should be clearly limited.	○	○	○	○
For general practitioners a detailed knowledge of osteopathy is useful.	○	○	○	○
I consider the partial coverage of costs for osteopathic treatments by the statutory health insurance companies to be justified.	○	○	○	○
The occupation "osteopath" should be established as an independent health profession in Germany.	○	○	○	○

Comments

Thank you for your cooperation!
