# Supplementary material for: Osteopathy in Germany: attitudes, beliefs and handling among general practitioners – results of a nationwide cross-sectional questionnaire survey
Source: BMC Fam Pract. 2021 Oct 7;22:197. doi: 10.1186/s12875-021-01545-2 (PMC8499418; doi:10.1186/s12875-021-01545-2)
Supplement: Supplementary file 2 — Additional file 2. Cross table with univariate predictors of recommendations (all items matched, ntotal = 341). [file 12875_2021_1545_MOESM2_ESM.pdf]

(2) Cross table with univariate predictors of recommendations (all items matched, ntotal=341)

| **Have you ever recommended patients to be treated by an osteopath? (dichotomized)** | | | | **in a few cases; no, never** | **yes, regulary; occasionally** | | |
|---|---|---|---|---|---|---|---|
| **Parameters** | *nvalid* | **Categories** | *ntotal (relative %)* | *absolute number (relative %)* | *absolute number (relative %)* | **p (Pearson Chi-Square)** | **Exp(B)/Odds Ratio (95% CI)** |
| Gender | 341 | male | 184 (54.0) | 69 (37.5) | 115 (62.5) | | |
| | | female | 157 (46.0) | 35 (22.3) | 122 (77.7) | **0.002** | **2.09 (1.29-3.38)** |
| Scientific qualification (PhD or habilitation?) | 316 | no | 113 (35.4) | 29 (25.7) | 84 (74.3) | | |
| | | yes | 203 (63.6) | 67 (33.0) | 136 (67.0) | 0.174 | 0.70 (0.42-1.17) |
| Age category | 338 | ≤ 39 years | 23 (6.7) | 7 (30.4) | 16 (69.6) | 0.997 | 1.00 (0.40-2.51) |
| | | 40-49 years | 70 (20.3) | 22 (31.4) | 48 (68.6) | | |
| | | 50-59 years | 134 (39.6) | 32 (23.9) | 102 (76.1) | | |
| | | ≥ 60 years | 111 (32.8) | 42 (37.8) | 69 (62.2) | | |
| In which year becoming medical specialist? | 321 | before 1980 | 9 (2.8) | 6 (66.7) | 3 (33.3) | | |
| | | 1980-1989 | 57 (17.8) | 20 (35.1) | 37 (64.9) | | |
| | | 1990-1999 | 103 (32.1) | 31 (30.1) | 72 (69.9) | | |
| | | 2000-2009 | 86 (26.8) | 26 (30.2) | 60 (69.8) | | |
| | | 2010-2019 | 66 (20.6) | 17 (25.8) | 49 (74.2) | 0.290 | 1.39 (0.76-2.56) |
| When established practice and started to work there? | 285 | before 1980 | 7 (2.5) | 5 (71.4) | 2 (28.6) | | |
| | | 1980-1989 | 38 (13.3) | 16 (42.1) | 22 (57.9) | | |
| | | 1990-1999 | 102 (35.8) | 31 (30.4) | 71 (69.6) | | |
| | | 2000-2009 | 77 (27.6) | 22 (28.6) | 55 (71.4) | | |
| | | 2010-2019 | 61 (21.4) | 17 (27.9) | 44 (72.1) | 0.301 | 1.31 (0.78-2.20) |
| Completed at least one additional qualification | 341 | no | 230 (67.5) | 70 (30.4) | 160 (69.6) | | |
| | | yes | 111 (32.5) | 34 (30.6) | 77 (69.4) | 0.971 | 0.99 (0.61-1.62) |
| Practice environment | 327 | big city | 72 (22.0) | 21 (29.2) | 51 (70.8) | 0.630 | 1.15 (0.65-2.04) |
| | | small city | 122 (37.3) | 38 (31.1) | 84 (68.9) | | |
| | | countryside | 133 (40.7) | 44 (33.1) | 89 (66.9) | | |
| Legal structure of practice | 267 | Single practice | 157 (58.8) | 48 (30.6) | 109 (69.4) | 0.494 | 1.20 (0.71-2.01) |
| | | Joint practice | 101 (37.8) | 37 (36.6) | 64 (63.4) | | |
| | | Medical care center | 9 (3.4) | 1 (11.1) | 8 (88.9) | | |
| Place of practice | 340 | States of former West Germany | 256 (75.3) | 86 (33.6) | 170 (66.4) | | |
| | | States of former East Germany + Berlin | 84 (24.7) | 17 (20.2) | 67 (79.8) | **0.021** | **1.99 (1.10-3.60)** |
| Do you know any osteopaths who work in the catchment area of your practice? | 337 | no | 76 (22.6) | 42 (55.3) | 34 (44.7) | | |
| | | yes | 261 (77.4) | 59 (22.6) | 202 (77.4) | **<0.001** | **4.11 (2.43-6.96)** |
| Do you know a qualified osteopath whom you would recommend to patients? | 328 | no | 112 (34.1) | 66 (58.9) | 46 (41.1) | | |
| | | yes | 216 (65.9) | 37 | 179 | **<0.001** | **6.94 (4.14-11.64)** |
| General feedback of patients regarding their osteopathic treatment | 333 | rather/mainly positive | 229 (68.8) | 40 (17.5) | 189 (82.5) | **<0.001** | **7.26 (4.31-12.22)** |
| | | heterogeneous | 76 (22.8) | 42 (55.3) | 34 (44.7) | | |
| | | rather/mainly negative | 9 (2.7) | 7 (77.8) | 2 (22.2) | | |
| | | no feedback | 19 (5.7) | 14 (73.7) | 5 (26.3) | | |
| Have you ever been treated by an osteopath yourself? | 333 | no | 222 (66.7) | 90 (40.5) | 132 (59.5) | | |
| | | yes | 111 (33.3) | 13 (11.7) | 98 (88.3) | **<0.001** | **5.14 (2.72-9.72)** |
| How would you rate your knowledge of the concepts of osteopathy and its treatment techniques? | 338 | no/little expertise | 195 (57.7) | 71 (36.4) | 124 (63.6) | | |
| | | good/very good expertise | 143 (42.3) | 33 (23.1) | 110 (76.9) | **0.009** | **1.91 (1.17-3.14)** |
